# Trends and age-period-cohort effects on hypertension mortality rates from 1998 to 2018 in Mexico

**DOI:** 10.1038/s41598-021-96175-0

**Published:** 2021-09-02

**Authors:** Lilia V. Castro-Porras, Rosalba Rojas-Martínez, Carlos A. Aguilar-Salinas, Omar Yaxmehen Bello-Chavolla, Carlos Becerril-Gutierrez, Consuelo Escamilla-Nuñez

**Affiliations:** 1 Policies, Population and Health Research Center, Faculty of Medicine, Universidad Nacional Autónoma de México, Mexico City, Mexico; 2 Reproductive Health Department, Center for Population Health, Instituto Nacional de Salud Pública, Mexico City, Mexico; 3 Metabolic Diseases Research Unit, Instituto Nacional de Ciencias Médicas y Nutrición Salvador Zubiran, Mexico City, Mexico; 4 Department of Endocrinology and Metabolism, Instituto Nacional de Ciencias Médicas y Nutrición Salvador Zubiran, Mexico City, Mexico; 5 Tec Salud, Instituto Tecnologico y de Estudios Superiores de Monterrey, Monterrey, NL México; 6 Division of Research, Instituto Nacional de Geriatría, Mexico City, Mexico; 7 Environmental Health Department, Center for Population Health, Instituto Nacional de Salud Pública, Fray Pedro de Gante # 12, Belisario Domínguez Sección 16, Tlalpan, C. P. 14080 Mexico City, Mexico

**Keywords:** Health care, Disease prevention, Health policy, Public health

## Abstract

Arterial hypertension is a major global health problem. It is the main risk factor for preventable death and the leading cause of premature death in the world. This study aims to describe the changes in hypertension-related mortality in Mexico between 1998 and 2018. Using death certificates and national population public data sets, a total of 335,863 deaths due to hypertension were found in Mexico, disaggregated by sex and age, during the time period covered in this study. An age-period-cohort analysis was conducted to show trends in hypertension mortality rates. Mortality due to hypertension in Mexico affects more women than men. In the most recent cohorts, the risk of dying from hypertension is two times higher in men compared to women. Hypertensive kidney disease is found to be the main underlying cause, with an average increase throughout the period studied. Our results indicate that mortality rates due to hypertension continue to grow and point to an alarming trend of mortality shifting towards younger ages, with sex-based disparities in absolute numbers and in changing trends.

## Introduction

Arterial hypertension is a major global health problem^1–3^, and it is the main modifiable risk factor for preventable deaths^4,5^. In Mexico, hypertension is not one of the main causes of mortality; however, it is important to know how many people are affected each year and whether mortality has changed in recent years, mainly because mortality due to hypertension is considered a premature and avoidable death. In this sense, showing evidence of this avoidable problem could contribute to alerting those responsible for managing the health system^6^. The prevalence of arterial hypertension among Mexican adults in 2012 was 31.5%, and was higher among men than women (32.3% and 30.7% respectively). Of the total population with hypertension, 47.3% are unaware of their diagnosis (38.7% of women and 57.8% of men). Prevalence increases in direct proportion with age. Among people who are known to be hypertensive, 73.9% are in medical therapy for hypertension, and out of these, only 51.2% have controlled blood pressure^7^. Regionally, over the past four decades, the widespread use of antihypertensive drugs has kept the mean blood pressure constant^8^, despite the observed increase in prevalence. In low- and middle-income countries, the prevalence is nearly 31.5%, equivalent to 1.04 billion people, while in high-income countries, the prevalence is 28.5% (349 million people)^5^.

Hypertension diagnosis and treatment are heterogeneous in many developing countries. This is the case for Mexico, in which nearly 30% of the population does not have access to primary care. Medical services are provided by several governmental or private health providers that offer services with varying levels of quality. Services are paid out of pocket by a large percentage of the population.

In a study that considered 194 countries, including Mexico, it was reported that between 1990 and 2017, hypertension was responsible for the largest number of all-cause deaths, followed by smoking and high fasting plasma glucose^9^. A previous report on mortality trends in Mexico suggested that between 2000 and 2008, the age-adjusted mortality rate per 100,000 inhabitants increased from 15.7 to 18.5, being higher among women than men, and higher among people who did not complete primary school than those with more education^10^. To identify determinants which have impacted hypertension mortality rates in Mexico, the Age-Period-Cohort approach offers an advantage as it disentangles age, period and cohort-specific effects that allow for a better understanding of the potential modifiers of these trends, which could inform public policy to further reduce the burden of hypertension in Mexico^11^. Negative socio-economic changes and the growing trend in obesity prevalence cause a period or cohort effect, which was detectable with our analytical approach. However, the fitting and interpretation of age-period-cohort models requires great care because of the well-known identifiability problem that exists; given any two of the three factors of age, period, and cohort, the third is determined^12^. Here, we describe the evolution of mortality due to arterial hypertension in Mexico, considering the dimensions of age, period and year of birth, in order to conclude how they affected the mortality rates in the period from 1998 to 2018.

## Methods

### Mortality data

The number of deaths related to hypertension disaggregated by age and sex were obtained from the dynamic information cubes accessed through the website of the National Statistics and Geography Institute (*Instituto Nacional de Estadística y Geografía*; INEGI) for the period from 1998 to 2018^6^*.* This data set is comprised of systematic daily mortality records collected by the Ministry of Health, and summarizes the information on an annual basis using the tenth revision of the International Classification of Diseases (ICD-10) codes. For these analyses, we considered deaths attributable to hypertension as ICD-10 codes I10 (Essential or Primary Hypertension), I11 (Hypertensive Heart Disease), I12 (Hypertensive Kidney Disease), I13 (Hypertensive Heart Disease and Hypertensive Kidney Disease) and I15 (Secondary Hypertension). To standardize mortality data to create rates per 100,000 population, we used population data from 1998 to 2018, disaggregated by sex and age, obtained from the national population projections available on the website of the National Population Council (*Consejo Nacional de Población*; CONAPO, 2020)^13^. Sex-specific mortality rates were calculated and standardized by age employing the direct method^14^, using a standard population (e.g. the distribution by sex and age of the entire population of the country obtained from the Population and Housing 2010 Census^15^ and World Population^16^).

We considered disaggregated ages from 20 to 84 for the calculation of crude and adjusted mortality rates; for model generation, we used ages grouped into quintiles (13 groups).

### Mortality trends over time

To determine changes in mortality due to the specific and total causes of hypertensive disease over time, we obtained the annual percentage change and the average annual percentage change over the entire study period (AnPC and AAnPC), as well as 95% confidence intervals (95% CI) by sex, using a "*joinpoint*" regression analysis^17^. Age-adjusted mortality rates followed a Poisson distribution, and statistical significance was considered, allowing for a maximum of 4 joinpoints. For the analysis of age, period and cohort, a Poisson dose–response model proposed by Kuang and collaborators was used, where the Poisson family, log link and population as an offset were specified^18^. To solve the identification problem in the age, period, and cohort models, Kuang proposed a canonical parameterization based on the accelerations of trends in the three factors, facilitating interpretation, estimation and forecasting^18^.

### Age-period-cohort analyses

Age-Period-Cohort (APC) models were evaluated, sex-stratified, and their 95% confidence intervals were generated. Based on the length of the observation period, the median was selected as the reference point for the cohort and the period. Two outcomes were evaluated: mortality rates and the relative risk of death due to hypertension. To estimate the effects of age, period, and cohort, we selected the models with significant effects and better performance assessed using the Akaike Information Criterion (AIC), and the p-value associated with the deviance test (Ho: APC model vs. Restricted model).

Joinpoint analyses were performed with the Joinpoint Regression Program 4.8.01-April 2020^19^ and the age-period-cohort analysis was performed using the APC v1.3 package^20^ of the R software version 3.6.2.

## Results

### Hypertension-related mortality from 1998 to 2018

**Figure 1:**
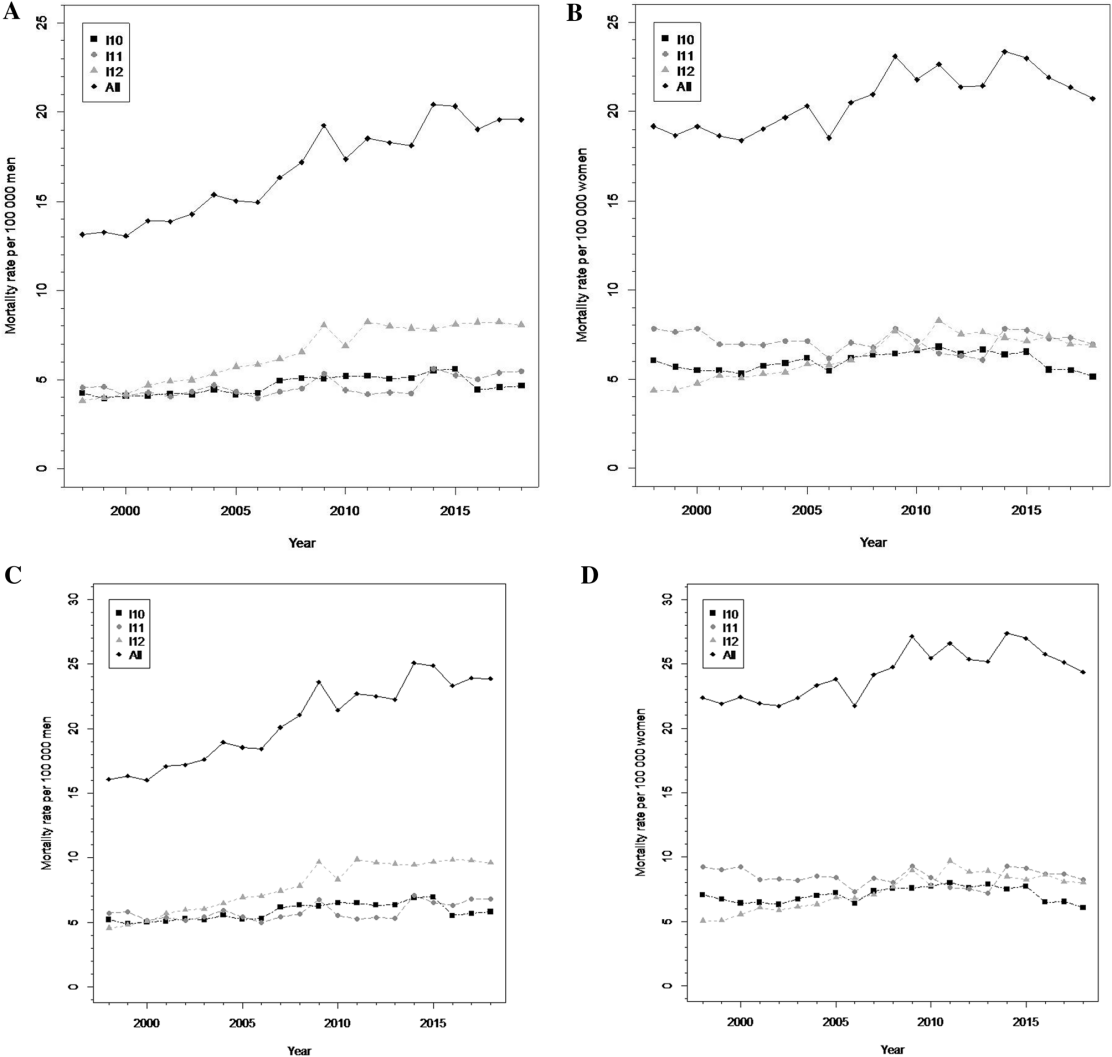
Mortality trends of hypertension and specific causes (per 100 000 population) by sex. Mexico, 1998–2018. (**A**) Men^a^. (**B**) Women^a^. (**C**) Men^b^. (**D**) Women^b^. I10, essential hypertension; I11, hypertensive heart disease; I12, hypertensive kidney disease; All, all underlying causes due to hypertension (I10, I11, I12, I13 and I15); ^a^Adjusted mortality rate by age from 2010 Mexican Census. ^b^Adjusted mortality rate by age from World Population (WHO 2000–2025).

In Mexico, a total of 335,863 deaths from hypertensive disease were recorded between 1998 and 2018. Annual deaths due to hypertension increased by 1.3% (95% CI 0.4, 2.2) per year. The growth in mortality rates was greater in men than in women (2.4% (95% CI 2.1, 2.8) versus 0.6% (95% CI − 0.3, 1.6), respectively). However, mortality rates during this period were greater in women than in men; in men, the highest rate reached 20 per 100,000, while in women it reached 23.5. Although we found different magnitudes, similar trends were observed when we used adjusted rates from the world population; in men, the highest rate reached 25 per 100,000 while in women it reached 27 (Fig. 1).

### Cause-specific hypertension-related mortality trends

The contribution of the underlying causes to total hypertension mortality was as follows: essential hypertension 31.1%, hypertensive heart disease 33.6%, hypertensive kidney disease 29.9%, hypertensive heart and kidney disease 5.4%, and secondary hypertension 0.02%. In the case of essential hypertension, annual mortality rates between 1998 and 2004 grew by 1.6% (95% CI 1.0, 2.2); in contrast, between 2014 and 2018, the annual mortality rate decreased by 5.7% (95% CI − 10.1, − 1.2). We observed an annual increase of 5.2% (95% CI 4.5, 5.9) in mortality due to hypertensive kidney disease between 1998 and 2011, which was higher in men than in women (3.9% and 2.5% respectively). Finally, we observed an increase of 1.1% (95% CI 0.4, 1.7) in hypertensive heart disease mortality rates in men, but not in women (Table 1).

**Table 1:** Mortality (adjusted mortality rate by age) trends of hypertension by sex and specific cause (per 100,000 population), Mexico 1998–2018.

Specific cause	Segment 1		Segment 2		Segment 3		Full range 1998–2018	Last 10 years 2009–2018
	Period	AnPC (95% CI)	Period	AnPC (95% CI)	Period	AnPC (95% CI)	AAnPC (95% CI)	AAnPC (95% CI)
**Total**								
I10	1998–2014	1.6 (1.0, 2.2)	2014–2018	− 5.7 (− 10.1, − 1.2)			0.1 (− 0.9, 1.0)	− 5.7 (− 10.1, − 1.2)
I11	1998–2018	0.3 (− 0.3, 0.9)					0.3 (− 0.3, 0.9)	0.3 (− 0.3, 0.9)
I12	1998–2011	5.2 (4.5, 5.9)	2011–2018	− 0.4 (− 2.1, 1.2)			3.2 (2.5, 3.9)	− 0.4 (− 2.1, 1.2)
All	1998–2015	2.0 (1.6, 2.4)	2015–2018	− 2.5 (− 8.2, 3.4)			1.3 (0.4, 2.2)	− 1.4 (− 5.4, 2.7)
**Men**								
I10	1998–2014	2.0 (1.4, 2.7)	2014–2018	− 4.8 (− 9.9, 0.5)			0.6 (− 0.5, 1.8)	− 1.1 (− 3.3, 1.2)
I11	1998–2018	1.1 (0.4, 1.7)					1.1 (0.4, 1.7)	1.1 (0.4, 1.7)
I12	1998–2011	5.8 (5.1, 6.5)	2011–2018	0.4 (− 1.4, 2.1)			3.9 (3.1, 4.6)	1.5 (0.3, 2.8)
All	1998–2018	2.4 (2.1, 2.8)					2.4 (2.1, 2.8)	2.4 (2.1, 2.8)
**Women**								
I10	1998–2000	− 5.6 (− 17.7, 8.2)	2000–2013	1.8 (1, 2.7)	2013–2018	− 5.4 (− 8.2, − 2.5)	− 0.8 (− 2.2, 0.7)	− 2.2 (− 3.8, − 0.7)
I11	1998–2018	− 0.2 (− 0.8, 0.4)					− 0.2 (− 0.8, 0.4)	− 0.2 (− 0.8, 0.4)
I12	1998–2011	4.6 (3.9, 5.3)	2011–2018	− 1.4 (− 3, 0.3)			2.5 (1.8, 3.2)	− 0.1 (− 1.3, 1.1)
All	1998–2015	1.4 (0.9, 1.8)	2015–2018	− 3.5 (− 9.4, 2.8)			0.6 (− 0.3, 1.6)	− 0.3 (− 2.2, 1.7)

*AnPC* annual percent change, *AAnPC* average annual percent change, *I10* essential hypertension, *I11* hypertensive heart disease, *I12* hypertensive kidney disease, *All* all underlying causes due to hypertension (I10, I11, I12, I13 and I15).

### Sex and age-specific mortality rates due to hypertension

**Figure 2:**
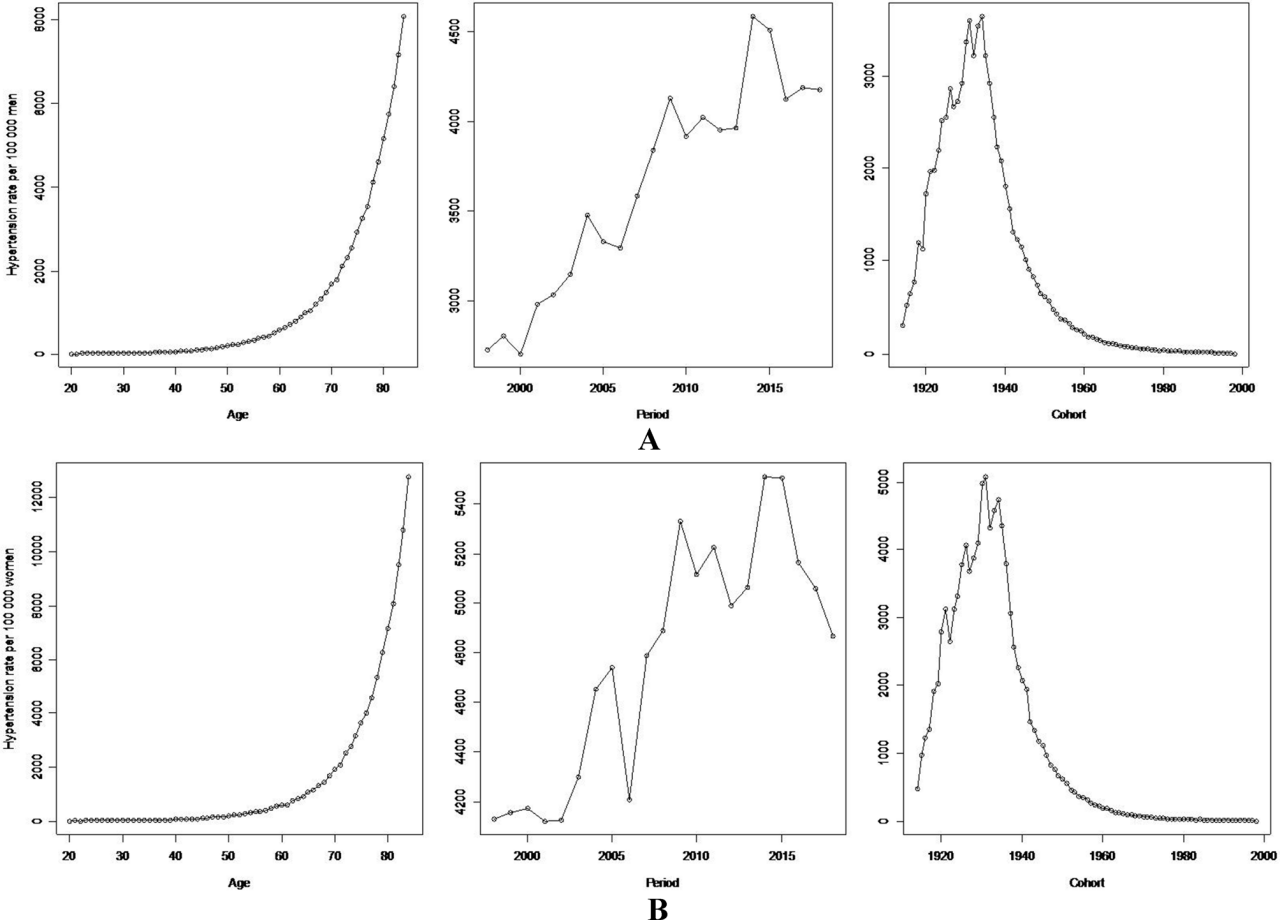
Hypertension mortality rate by age, period and cohort. Mexico, 1998–2018. (**A**) Men. (**B**) Women.

**Figure 3:**
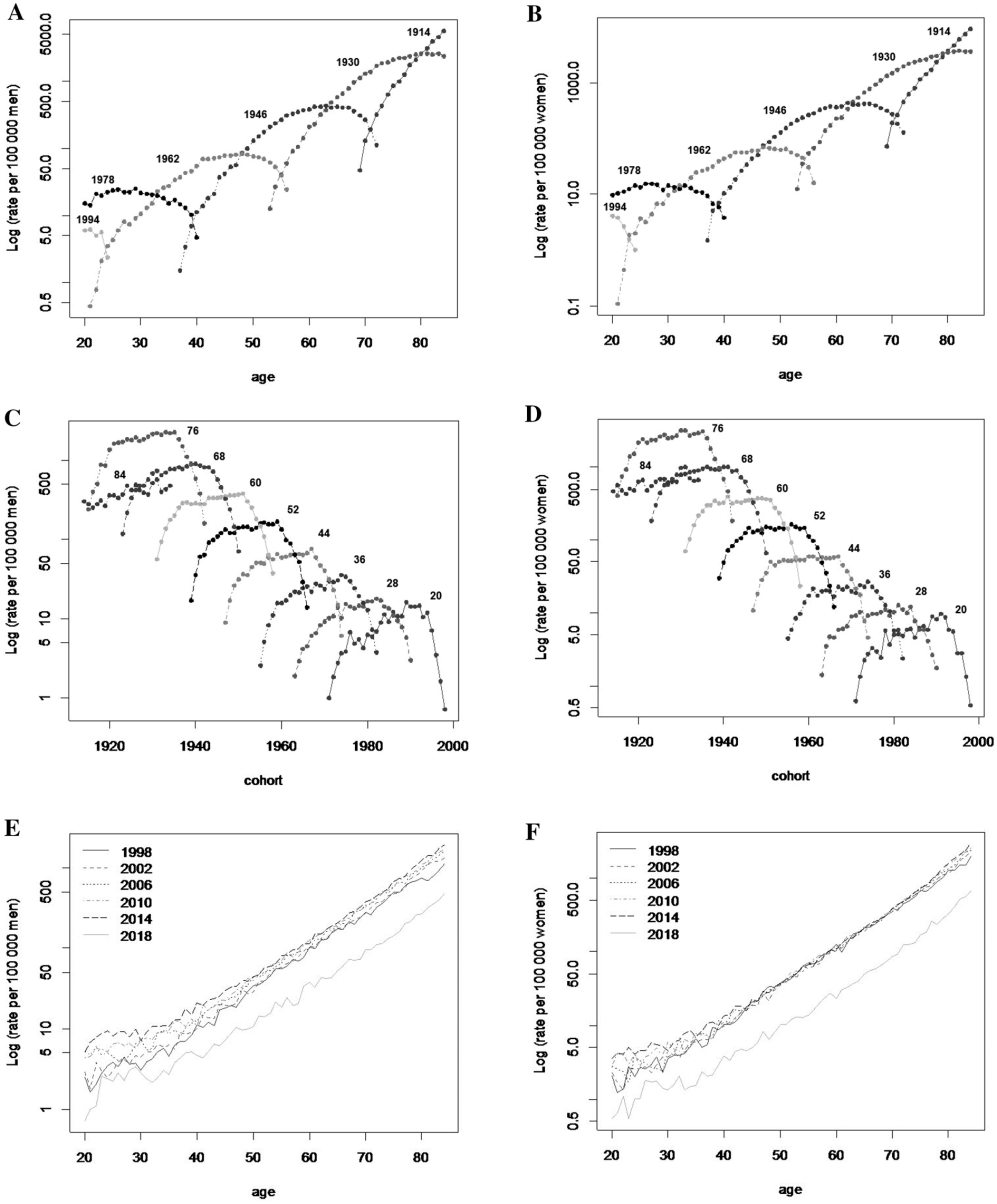
Hypertension mortality curves by age-specific within birth-year cohort and sex. Mexico, 1998–2018. Age-specific within cohort (**A**) Men, (**B**) Women. Birth-year cohort within age-specific (**C**) Men, (**D**) Women. Age-specific within period (**E**) Men, (**F**) Women.

For both sexes, the number of deaths attributable to hypertension increased in cases of those older than 60. Annual mortality rates by year of death showed an increase among men, while among women there were abrupt reductions (2006 and 2018). Finally, when analyzing by birth-year, the greatest number of deaths in both sexes were recorded in the 1930 and 1940 birth-year cohorts (Fig. 2). Overall, mortality rates increased with age for all hypertension-related deaths within a particular birth-year cohort. When comparing the cohort born between 1962 and 1945 with the cohort born between 1946 and 1929, the average ages at death were 38.5 ± 7.6 and 54.5 ± 7.6 respectively, and mortality rates reached its peak at 45 and 62 years of age in such cohorts. In the more recent birth-year cohorts, age-specific hypertension mortality rates increased. Finally, for both genders there has also been an overall increase in the mortality rate by age during this time period; men showed a greater variability in rates than women (Fig. 3).

### APC models

From the graphical representation of the crude effects, we observed that the simplified models (age-period or age-cohort) could explain the data (Fig. 3). We then compared each 2-factor model with the 3-factor model (APC model) using the likelihood ratio test. We observed that the 2-factor models were similar to the 3-factor model (Supplementary Fig. 1), so we chose the 2-factor model based on the Akaike Criterion. It is important to highlight that, although the three-factor model generally tends to fit better, most of the time those analyses are not conclusive in deciding whether the observed trend is due to a period or cohort effect.

**Figure 4:**
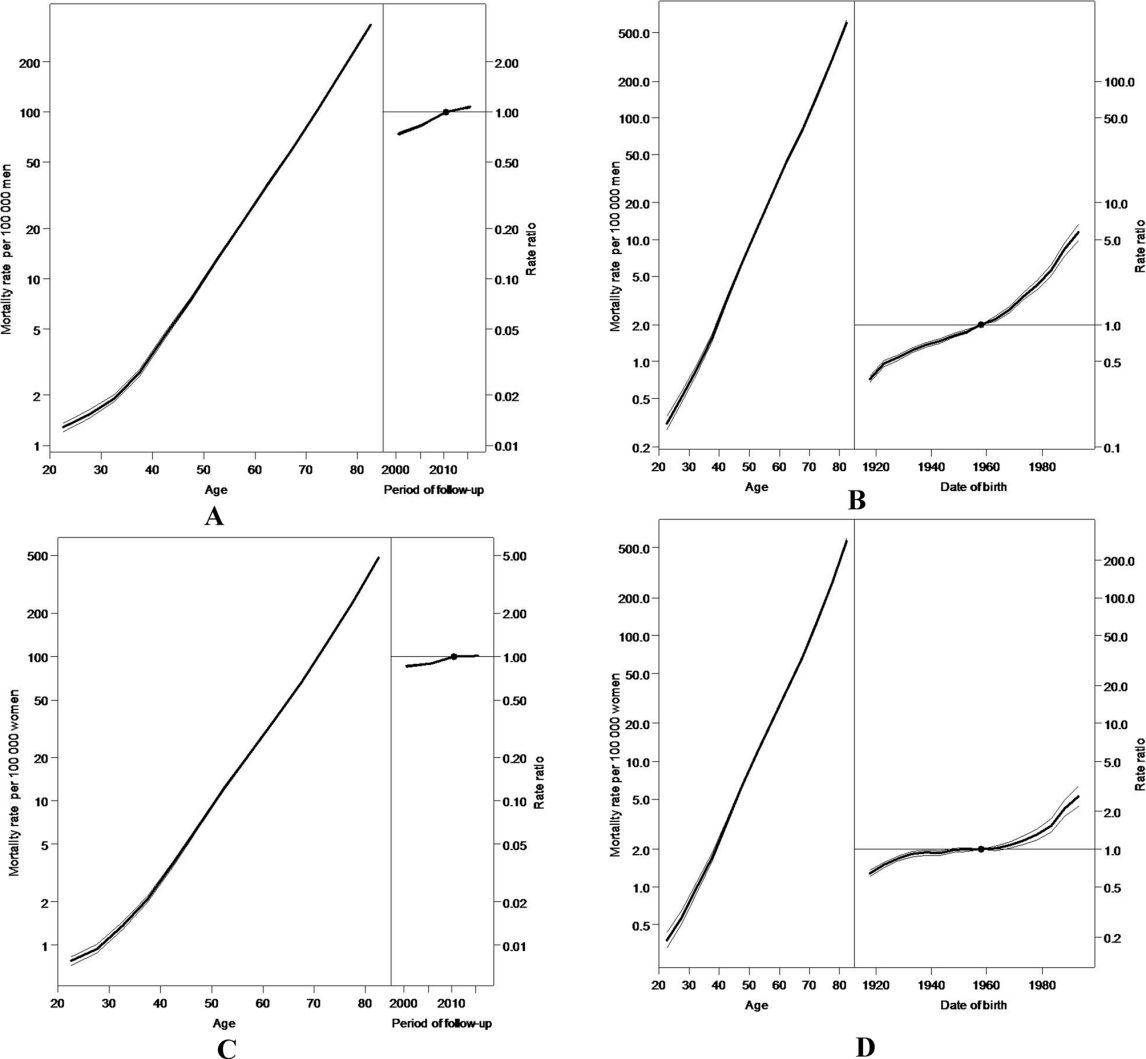
Age, period and cohort effect on hypertension mortality by sex. Mexico, 1998–2018. Age-specific rates and rate-ratios relative to the period 2008–2013 (**A**) Men, (**C**) Women. Age-specific rates and rate-ratios relative to the cohort 1958–1963 (**B**) Men, (**D**) Women.

For both AP and AC models, mortality rates increased with age by 1.0% (95% CI 1.0, 1.01) on average each year for both the reference period (2008) and the cohort (1958), independent of sex. For the AP model, the relative risk of death attributable to hypertension increased in men, with an almost neutral risk in women. For the AC model, the relative risk estimates were greater for the most recent birth year cohorts as compared to the reference cohort. The greatest risk was reported in the 1994 cohort as compared to the reference birth-year cohort, with RR estimates higher in men as compared to women ($RR\approx 6$ versus $RR\approx 2.5$) (Fig. 4 and Supplementary Tables 1 and 2).

## Discussion

In this study, we confirm previous reports that indicate that mortality trends in Mexico have steadily increased over time. When disaggregating by underlying causes of hypertension using an APC approach, we observed that essential hypertension-related mortality has decreased in recent years, whilst hypertensive kidney disease increased more in men as compared to women, and hypertensive cardiovascular disease-related mortality rose only in men. The APC approach allows us to distinguish the effect of age on mortality due to hypertension, regardless of the birth-year or period in which death occurs. This in turn allows us to understand the change in mortality rates attributable to aging, despite changes in overall population structure. Our results provide an overview of these trends in Mexico and allow us to explain how mortality due to hypertension has changed at different ages, cohorts, and periods.

Hypertension is the leading cause of cardiovascular disease and premature death worldwide. It is associated with shorter life expectancy overall, and more years lived with CVD^21,22^. In recent years, hypertension prevalence has increased differentially across countries, with a higher prevalence in low-income countries, where the main discrepancies are due to education level and the quality of health services^5,22^. Many studies have focused on hypertension as morbidity; however, few have studied mortality from this cause. Of the 722,611 deaths reported in Mexico from all causes in 2018^6^, hypertension contributed 3.3%, and our results showed that the age of death has been advancing each year, as well as showing that hypertension-related mortality in Mexico has increased in the last 20 years, with a greater rise in men than in women, as has been previously reported^10^. This increase could be explained by the interaction with other cardio-metabolic diseases including diabetes, or to intrinsic sex-based differences in blood pressure management in Mexico (45.6%), which is lower overall compared to other countries^23^. Systematic differences attributable to improved management of hypertension and increased adherence to treatment could influence this disparity between countries^24^. It is worth noting, however, that this pattern is not universal, as there are high-income countries with adequate health systems, such as the United States, which also show an increase in mortality rates^2^. In Mexico, universal coverage is still a work in progress. The proportion of patients that effectively received care after seeking services from the health system to control the disease does not go above 50% of the service users^25^. If all of the hypertension cases had been treated, the cost at the national level would have doubled, from 3.2 billion dollars to 7.4 billion dollars. An increase in the out-of-pocket expenses due to hypertension treatment was observed in the time period from 2005 to 2015^25^. This highlights the importance of health promotion related to preventable diseases such as hypertension.

Death certificates consider five underlying causes of death due to hypertension. One of them, hypertensive kidney disease, has been highlighted because of a recent increase in mortality attributable to Chronic Kidney Disease^9^. A 2013 report in Mexico suggested a rise in standardized mortality rates of hypertensive kidney disease during the period from 1998 to 2009, from 3.35 per 100,000 inhabitants to 6.74, with a notable increase from the age of 50^26^. Our findings are consistent with these findings for the 1998 to 2018 period, with a further sex-based discrepancy in mortality rates, being greater in men than in women. Notably, we also report an increase in hypertensive heart disease-related mortality in men, which falls in line with an excessive risk for hypertension-related cardiovascular mortality in men^27^.

We observed an age effect. Our results indicate that mortality rates due to hypertension were higher as age increased from 40, with a higher increase after 55 in both men and women. These results are in line with what was previously reported in a study of the United States conducted between 2011 and 2016, in which mortality rates due to hypertension increased at 35 with a higher increase after 55^2^. This could be explained by the marked increase in hypertension as age increases from 30 onwards in Mexico, and the minimal management of blood pressure resulting from various causes, including undiagnosed hypertension, therapeutic failure or the presence of concomitant diseases^5,24^. Furthermore, we found a cohort effect. In recent cohorts, we observed an overall increase in the mortality rate. This could be explained by an shift in the incidence of hypertension to younger populations, largely attributable to an increase in unhealthy lifestyle linked to calorie-enriched diets and the consumption of alcohol and tobacco^28^. We also report the cohort effect of an increased risk of hypertension-related mortality in the most recent cohorts, higher in men than in women. A similar sex-based cohort effect was reported for hypertension in China, in which the cohort effect was observed as being stronger among men than women^29^. In Mexico, this could be related to the fact that more than half of the adults with undiagnosed hypertension are men^24^. Additionally, overweight and obesity are a highly prevalent problem in the Mexican population in all age groups, in all regions of the country and in urban and rural areas. It has been reported that one in three school-age children, about 35% of adolescents and more than three quarters of adults present these conditions^30^.

Although mortality due to hypertension has increased or even decreased in some years during the study period, this does not mean that the prevalence of hypertension has decreased, but rather that the impact of factors such as medical treatment, treatment adherence or preventive health programs may have a positive effect in reducing overall mortality due to hypertension^31,32^. On the other hand, increased mortality due to the underlying cause of kidney disease and cardiovascular risks may be related to increased salt intake or to an increase in the incidence of chronic kidney disease due to cardio-metabolic comorbidities^31,33^. In 2013, the World Health Assembly agreed on nine voluntary global goals for the prevention and control of non-communicable diseases and a target to reduce the average salt intake by 30% by 2025^34^. Our results showed an effect in the period studied; however, since causes are etherogeneous, we do not have sufficient evidence to associate this effect to any specific cause.

It is important to highlight that our results are based on the documentation of the underlying causes of death on death certificates, which may be prone to bias and unreliability. Failures in the completion of the death certificates could have interfered with the appropriate coding of the death cause, due to the fact that physicians sometimes downplay the importance of the sequence of events leading to death^35^. In addition, our results could have been affected by delay or misclassification bias due to recording the final event of death on a death certificate, for example, a heart attack, rather than the leading cause of death. Additionally, the lack of clinical and socioeconomic background data limited the analysis.

In conclusion, we observed that from 1998 to 2018, age-adjusted mortality rates due to hypertension were higher in women than compared to men. Similarly, the risk of hypertension-related mortality was doubled in men compared to women in recent cohorts. Among the underlying causes of mortality due to hypertension, hypertensive kidney disease has become the leading underlying cause of death with an average growth over the entire study period and a higher impact among men. Our results show that mortality rates due to hypertension continue to grow and indicate an alarming trend of mortality shifting towards younger ages, with sex-based disparities in absolute numbers and in changing trends.

## Supplementary Information

Supplementary Information.

Supplementary Figure 1.

Supplementary Figure 2.
