# Supplementary material for: Trends and age-period-cohort effects on hypertension mortality rates from 1998 to 2018 in Mexico
Source: Sci Rep. 2021 Sep 2;11:17553. doi: 10.1038/s41598-021-96175-0 (PMC8413460; doi:10.1038/s41598-021-96175-0)
Supplement: Supplementary file 1 — Supplementary Information. [file 41598_2021_96175_MOESM1_ESM.docx]

**Table 1.** Age-specific rates and rate-ratios relative to the period 2008-2013.

| Period | Men  RR(95%CI) | Women  RR(95%CI) |
| --- | --- | --- |
| 1998 | 0.740(0.725, 0.754) | 0.855(0.840, 0.870) |
| 2003 | 0.837(0.822, 0.852) | 0.892(0.877, 0.906) |
| Reference period 2008 | **1** | **1** |
| 2013 | 1.076(1.060, 1.093) | 1.009(0.994, 1.024) |

RR: Relative risk and 95%CI: 95% Confidence Interval. Age-period model.

**Table 2.** Age-specific rates and rate-ratios relative to the cohort 1958-1963.

| Cohort | Men  RR(95%CI) | Women  RR(95%CI) |
| --- | --- | --- |
| 1918 | 0.359(0.337, 0.382) | 0.642(0.606, 0.679) |
| 1923 | 0.482(0.457, 0.509) | 0.751(0.714, 0.790) |
| 1928 | 0.540(0.513, 0.567) | 0.844(0.804, 0.886) |
| 1933 | 0.618(0.589, 0.648) | 0.912(0.870, 0.956) |
| 1938 | 0.684(0.654, 0.717) | 0.939(0.897, 0.983) |
| 1943 | 0.732(0.701, 0.765) | 0.932(0.893, 0.974) |
| 1948 | 0.821(0.788, 0.855) | 0.980(0.941, 1.021) |
| 1953 | 0.882(0.849, 0.917) | 0.995(0.957, 1.034) |
| Reference cohort 1958 | **1** | **1** |
| 1963 | 1.120(1.071, 1.171) | 1.013(0.966, 1.061) |
| 1968 | 1.333(1.260, 1.411) | 1.071(1.008, 1.137) |
| 1973 | 1.712(1.595, 1.838) | 1.174(1.086, 1.269) |
| 1978 | 2.123(1.942, 2.321) | 1.317(1.192, 1.455) |
| 1983 | 2.808(2.527, 3.121) | 1.539(1.363, 1.739) |
| 1988 | 4.151(3.674, 4.689) | 2.109(1.823, 2.439) |
| 1993 | 5.728(4.924, 6.662) | 2.645(2.197, 3.185) |

RR: Relative risk and 95%CI: 95% Confidence Interval. Age-cohort model

**Figure 1**. Age, period and cohort effect on hypertension mortality by sex. Mexico, 1998-2018

**Figure. Men Figure. Women**
