# Supplementary figures and images for: Trends and age-period-cohort effects on hypertension mortality rates from 1998 to 2018 in Mexico
Source: Sci Rep. 2021 Sep 2;11:17553. doi: 10.1038/s41598-021-96175-0 (PMC8413460; doi:10.1038/s41598-021-96175-0)

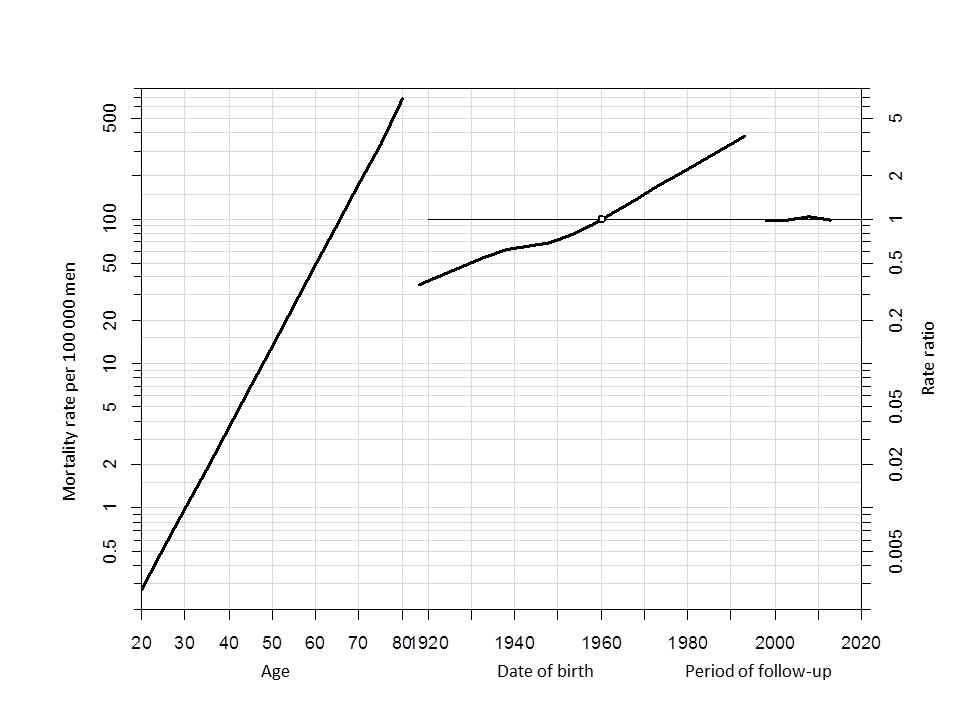

Supplement: Supplementary file 2 — Supplementary Figure 1. [file 41598_2021_96175_MOESM2_ESM.tif]

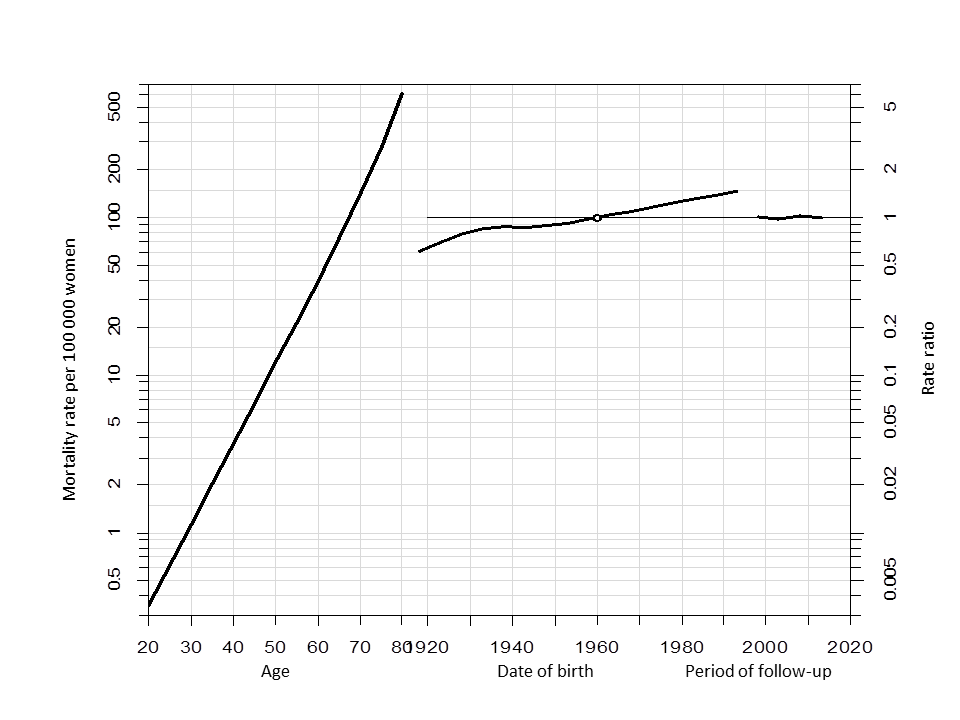

Supplement: Supplementary file 3 — Supplementary Figure 2. [file 41598_2021_96175_MOESM3_ESM.tif]
